# Association between physical-activity trajectories and cognitive decline in adults 50 years of age or older

**DOI:** 10.1017/S2045796021000688

**Published:** 2021-12-27

**Authors:** Boris Cheval, Zsófia Csajbók, Tomáš Formánek, Stefan Sieber, Matthieu P. Boisgontier, Stéphane Cullati, Pavla Cermakova

**Affiliations:** 1 Swiss Center for Affective Sciences, University of Geneva, Geneva, Switzerland; 2 Laboratory for the Study of Emotion Elicitation and Expression (E3Lab), Department of Psychology, University of Geneva, Geneva, Switzerland; 3 National Institute of Mental Health, Klecany, Czech Republic; 4 Second Faculty of Medicine, Charles University, Prague, Czech Republic; 5 Faculty of Science, Charles University, Prague, Czech Republic; 6 EpiCentre, Department of Psychiatry, University of Cambridge, Cambridge, UK; 7 LIVES Centre, Swiss Centre of Expertise in Life Course Research, Carouge, Switzerland; 8 Center for the Interdisciplinary Study of Gerontology and Vulnerability, University of Geneva, Geneva, Switzerland; 9 School of Rehabilitation Sciences, Faculty of Health Sciences, University of Ottawa, Ottawa, Canada; 10 Bruyère Research Institute, Ottawa, Canada; 11 Population Health Laboratory, University of Fribourg, Fribourg, Switzerland; 12 Department of Readaptation and Geriatrics, University of Geneva, Geneva, Switzerland

**Keywords:** Aging, cognition, longitudinal studies, physical activity

## Abstract

**Aims:**

To investigate the associations of physical-activity trajectories with the level of cognitive performance (CP) and its decline in adults 50 years of age or older.

**Methods:**

We studied 38 729 individuals (63 ± 9 years; 57% women) enrolled in the Survey of Health, Ageing and Retirement in Europe (SHARE). Physical activity was self-reported and CP was assessed based on immediate recall, verbal fluency and delayed recall. Physical-activity trajectories were estimated using growth mixture modelling and linear mixed-effects models were used to investigate the associations between the trajectories and CP.

**Results:**

The models identified two trajectories of physical activity: constantly high physical activity (*N* = 27 634: 71%) and decreasing physical activity (*N* = 11 095; 29%). Results showed that participants in the decreasing physical-activity group exhibited a lower level of CP compared to the high physical-activity group (immediate recall: ß = 0.94; 95% confidence interval [CI] = 0.92–0.95; verbal fluency: ß = 0.98; 95% CI = 0.97–0.98; delayed recall: ß = 0.95; 95% CI = 0.94–0.97). Moreover, compared with participants in the constantly high physical-activity group, participants in the decreasing physical-activity group showed a steeper decline in all cognitive measures (immediate recall: ß = −0.04; 95% CI = −0.05 to −0.04; verbal fluency: ß = −0.22; 95% CI = −0.24 to −0.21; delayed recall: ß = −0.04; 95% CI = −0.05 to −0.04).

**Conclusions:**

Physical-activity trajectories are associated with the level and evolution of CP in adults over 50 years. Specifically, our findings suggest that a decline in physical activity over multiple years is associated with a lower level and a steeper decline in CP.

## Introduction

Physical activity (PA) and cognitive performance (CP) are strongly linked and have been shown to decline with ageing (Cheval *et al*., 2018*b*, 2020*c*; Sebastiani *et al*., 2020). Multiple observational and interventional studies have demonstrated that a higher level of PA is associated with better CP in old age (Colcombe and Kramer, 2003; Angevaren *et al*., 2008; Hamer *et al*., 2018; Cheval *et al*., 2020*b*) However, most of these studies focused on the association between PA levels and CP. Yet, disregarding the intra-individual evolution of PA over time may have biased the observed association with CP (Mok *et al*., 2019). Because PA is a complex behaviour that evolves over time (Mok *et al*., 2019), examining the associations between life-course PA trajectories and CP is warranted.

Recent studies have attempted to address this gap by considering the evolution of PA over time (Wannamethee *et al*., 1998; Gregg *et al*., 2003; Stessman *et al*., 2009; Almeida *et al*., 2014; Jefferis *et al*., 2014; Elhakeem *et al*., 2018; Laddu *et al*., 2018; Saint-Maurice *et al*., 2019; Aggio *et al*., 2020; Sanchez-Sanchez *et al*., 2020). However, most of those studies were based on conventional methods to identify groups with different PA trajectories, which typically use clinical or empirical cut-points (Wannamethee *et al*., 1998; Gregg *et al*., 2003; Stessman *et al*., 2009; Almeida *et al*., 2014; Jefferis *et al*., 2014; Elhakeem *et al*., 2018). To reduce the reliance on these cut-points, data-driven approaches such as growth mixture modelling have been proposed (Nagin and Odgers, 2010). These models do not require a priori trajectory classifications as the trajectories emerge from the data (Aggio *et al*., 2020; Formánek *et al*., 2020). Using this approach (Barnett *et al*., 2008; Laddu *et al*., 2017), studies have shown PA trajectories in older adults were associated with multiple health outcomes including disability, cardiovascular diseases and all-cause mortality (Laddu *et al*., 2018, 2020; Saint-Maurice *et al*., 2019; Aggio *et al*., 2020; Sanchez-Sanchez *et al*., 2020). However, the associations of PA trajectories with CP and is unknown.

In this study, we aimed to investigate the associations of PA trajectories with CP level and its decline in adults 50 years of age or older. We hypothesised that unfavourable profiles (e.g., individuals with decreasing PA over time) would be associated with lower levels and steeper decline of CP compared to more favourable profiles (e.g., individuals maintaining high PA levels).

## Methods

### Design and population

Data were drawn from the Survey of Health, Ageing and Retirement in Europe (SHARE), an ongoing population-based study of health, social network and economic conditions of community-dwelling middle-aged and older individuals, living in 28 European countries and Israel (*N* = 139 556). SHARE was described in detail elsewhere (Börsch-Supan *et al*., 2013). Briefly, participants were sampled based on probability selection methods. Individuals eligible for the study were people aged 50 years or older and their partners, irrespective of age. Data were collected using computer-assisted personal interviewing (CAPI) in participants' homes. The study was initiated in 2004 and followed by six subsequent waves with approximately 2-year intervals and wave 7 being completed in 2017. Wave 3 was devoted to data collection related to childhood histories (SHARELIFE). This wave did not contain any data related to PA and CP and was therefore not used in the current study. This study was carried out in accordance with the Declaration of Helsinki. SHARE has been approved by the Ethics Committee of the University of Mannheim (waves 1–4) and the Ethics Council of the Max Plank Society (waves 4–7). All participants provided written informed consent. Data were pseudo-anonymised and participants were informed about the storage and use of the data and their right to withdraw consent.

**Fig. 1.**
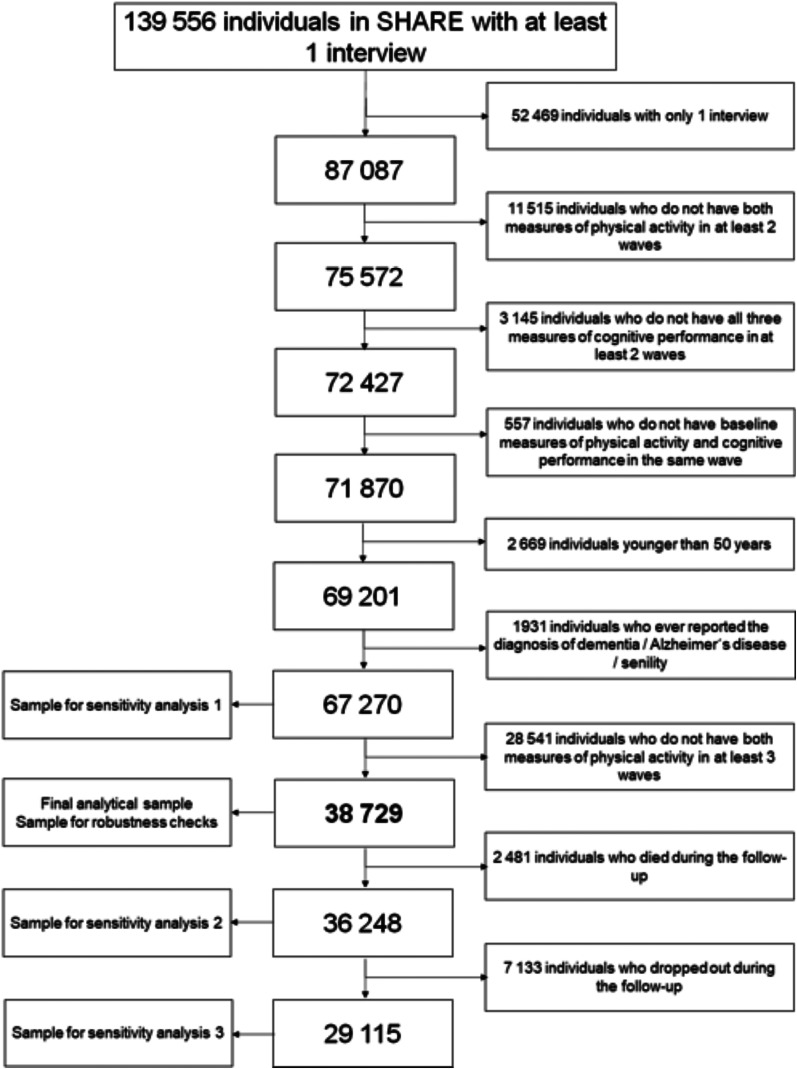
Selection of the study sample.

In the present study, we included a total of 38 729 participants who fulfilled the following criteria: (1) age = 50 years or older at baseline, (2) measures of PA in at least three waves, (3) measures of cognition in at least two waves and (4) no report of diagnosed dementia (Fig. 1).

### Measures

#### Physical activity

PA was assessed as part of CAPI in waves 1, 2, 4, 5, 6 and 7 using two questions. ‘*How often do you engage in vigorous physical activity, such as sports, heavy housework, or a job that involves physical labour?’* and ‘*How often do you engage in activities that require a low or moderate level of energy such as gardening, cleaning the car, or doing a walk?’* Participants answered on a four-point scale (0 = *Hardly ever, or never*; 1 = *One to three times a month*; 2 = *Once a week*; 3 = *More than once a week*). An overall score of PA ranging from 0 to 6 was created by summing up the scores on the two questions, with higher scores reflecting greater PA.

#### Cognitive tests

CP was assessed in waves 1, 2, 4, 5, 6 and 7 using validated tests of verbal fluency, immediate recall and delayed recall. In the verbal fluency test (Rosen, 1980), participants were instructed to name as many different animals as they could think of in 1 minute. The score was the total number of correctly named animals, with a higher score indicating higher verbal fluency. Immediate and delayed recall were assessed using an adapted 10-word delayed-recall test (Harris and Dowson, 1982). In the immediate-recall test, participants first listened to a 10-word list that was read out loud by the interviewer. Then, immediately after the reading of this list, participants were asked to recall as many words as possible. At the end of the cognitive testing session, the participants were asked again to recall any of the words from the list, which captured the delayed recall score. Both scores ranged from 0 to 10, with a higher score indicating greater performance.

### Covariates

Models were adjusted for sociodemographic and health-related characteristics identified as potentially confounding and mediating factors in the association between PA and CP level and decline (Trost *et al*., 2002; Kirk and Rhodes, 2011; Bauman *et al*., 2012; Choi *et al*., 2019; Cheval *et al*., 2021). The value of the covariates was the one at baseline if all PA and CP data were available. In case of missing data at baseline, the value of the covariate was taken from the closest available wave. The selected sociodemographic factors were area of Europe (Western Europe, Scandinavia, Southern Europe, Central and Eastern Europe), age (years), sex (male, female), education (seven categories based on the International Standard Classification of Education 1997), (United Nations Educational, 2006) residence (big city, suburbs or outskirts of a big city, large town, small town, rural area or village), household size (number of members), a partner in a household (yes, no), household net worth (standardised difference between household gross financial assets and financial liabilities), current job situation (working, not working), number of children, number of grandchildren and attrition (non-participation in all the waves or death during the follow-up; yes, no). Health-related characteristics were the number of limitations in instrumental activities of daily living (IADL), number of depressive symptoms assessed with the EURO-D scale (Prince *et al*., 1999), number of chronic diseases, body mass index (continuous variable), mobility limitations index (number of limitations), smoking (ever smoked daily, never smoked daily), alcohol use (more than two glasses of alcohol almost every day, less), frequency of eating fruits and vegetables (every day, 3–6 times a week, twice a week, once a week, less than once a week).

### Statistical analyses

#### PA trajectories

As in our previous study on trajectories of depressive symptoms (Formánek *et al*., 2020), growth mixture modelling with maximum likelihood estimation was used to identify latent trajectories of PA (Jung and Wickrama, 2008). This approach estimates latent classes following similar trajectories of PA over time with a high probability. Consistent with previous guidelines and literature (Van De Schoot *et al*., 2017; Formánek *et al*., 2020), we first freely estimated time slopes in a latent basis growth model that was entered to the growth mixture model (i.e., the classification). Second, the most parsimonious model among those with a different number of PA trajectories (i.e., classes) was determined using the following indicators: Akaike information criterion, Bayesian information criterion, sample-size adjusted Bayesian information criterion, Vuong–Lo–Mendell–Rubin likelihood ratio test, Luo–Mendell–Rubin adjusted likelihood ratio test and bootstrap likelihood ratio test (see Supplementary Methods). Finally, we checked the number of individuals within each PA trajectory group to ensure an adequate sample size.

#### Association between PA trajectories and baseline characteristics

First, independent samples *t*-tests and *χ*^2^ tests were used to compare the differences in baseline characteristics between PA trajectories. Second, a multivariable analysis using logistic regression was performed to estimate the odds ratio (OR) with a 95% confidence interval (CI) for the association between participants´ baseline characteristics and PA trajectories.

#### Association between PA trajectories and the level and rate of decline of CP

We used linear mixed-effects models to investigate the associations of PA trajectories with the level of CP and its rate of decline over time. The models included time in years since baseline, PA trajectory, their interaction term (PA trajectory × time) and covariates. Model 1 was adjusted for age and sex. Other sociodemographic covariates (birth cohort, region, education, residence, household size, partner in household, household net worth, current job situation, number of children, number of grandchildren and attrition) were included in Model 2. Health-related characteristics (limitations in IADL, depressive symptoms, number of chronic diseases, body mass index, mobility limitations index, smoking, alcohol use and eating behaviour) were included in Model 3. We adjusted for covariates group-wise in three steps to assess whether sociodemographic and health-related characteristics may explain the observed associations. The model random structure encompassed random intercepts for participants and random linear slopes for time. In addition, we stratified the dataset according to PA trajectories, and (1) fitted a crude model containing only time and (2) Models 1–3 (except interaction term) for each cognitive test, separately. Based on the fit of the crude stratified model, we visualised the cognitive decline across trajectories of PA and per cognitive tests.

#### Sensitivity and robustness analyses

We performed three sensitivity analyses (SA), in which we replicated the growth mixture modelling on (1) a sample of participants having at least two measures of PA, two measures of CP and no diagnosis of dementia (SA1: *N* = 67 270), (2) a sample only including surviving participants (SA2: *N* = 36 248) and (3) a sample of participants who neither dropped out nor died during the follow-up of the study (SA3: *N* = 29 115). We conducted two robustness analyses, in which we performed the growth mixture modelling on (a) the moderate physical activity measure only and (b) on the vigorous physical activity measure only.

### Data availability

Access to the SHARE data is provided free of charge on the basis of a release policy that gives quick and convenient access to all scientific users worldwide after individual registration. All details about the application and registration process can be found on this website: http://www.share-project.org. The study protocol and syntax of the statistical analysis will be shared upon request from the corresponding author of this study.

## Results

### PA trajectories

**Fig. 2.**
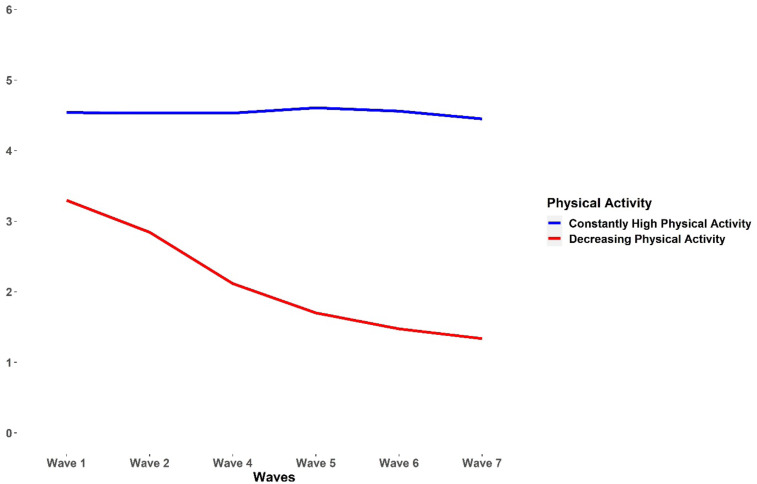
Physical activity trajectories.

The study sample included 38 729 adults 50 years of age or older (63 years on average, 57% women). Two PA trajectory groups emerged from the growth mixture modelling as the best model for describing the longitudinal data: constantly high PA (*N* = 27 634: 71%) and decreasing PA (*N* = 11 095; 29%) (Fig. 2). This two-trajectory solution showed an acceptable fit to the data, although with relatively low entropy (0.603), thereby indicating some overlap between the two classes. Results from the process of model selection, including the results of the sensitivity and robustness analyses, are provided in the supplementary materials.

### PA trajectories and baseline characteristics of the participants

Table 1 summarises the characteristics of the participants stratified by PA trajectory. Compared with the constantly high PA group, individuals with decreasing PA showed lower baseline level in the three measures of CP (mean verbal fluency 17.9 *v.* 21.5; mean immediate recall 4.7 *v.* 5.5; mean delayed recall 3.2 *v.* 4.1; *p*s < 0.001). In addition, individuals with decreasing PA were older (*p* < 0.001) and predominantly women (*p* < 0.001).

**Table 1.** Baseline characteristics of the participants across PA trajectories

	Constantly high physical activity (*n* = 27 634)	Decreasing physical activity (*n* = 11 095)	*p* Value
Cognition			
Immediate recall, mean ± s.d.	5.5 ± 1.6	4.7 ± 1.8	<0.001
Verbal fluency, mean ± s.d.	21.5 ± 7.3	17.9 ± 7.1	<0.001
Delayed recall, mean ± s.d.	4.1 ± 2.0	3.2 ± 2.0	<0.001
Sociodemographic characteristics			
Age, mean ± s.d.	61.2 ± 8.0	66.8 ± 9.6	<0.001
Women, *n* (%)	15 095 (55)	6819 (62)	<0.001
High education^a^, *n* (%)	6 601 (24)	1 487 (13)	<0.001
Highest decile of household net worth, *n* (%)	3 228 (12)	644 (6)	<0.001
Urban residence^b^, *n* (%)	7 667 (28)	3 061 (28)	0.76
Employed or self-employed, *n* (%)	11 166 (40)	1 750 (16)	<0.001
Two or more household members, *n* (%)	22 958 (83)	8 418 (76)	<0.001
Partner in household, *n* (%)	21 488 (78)	7 693 (69)	<0.001
Two or more children, *n* (%)	20 721 (75)	8 128 (73)	<0.001
Two or more grandchildren, *n* (%)	13 154 (48)	6 727 (61)	<0.001
Region, *n* (%)			
Western Europe	11 618 (42)	4 107 (37)	<0.001
Scandinavia	3 334 (12)	743 (7)	
Southern Europe	4 459 (16)	2 870 (26)	
Central and Eastern Europe	7 204 (26)	2 820 (25)	
Israel	1 019 (4)	555 (5)	
Health-related characteristics			
High depressive symptoms^c^, *n* (%)	5 909 (21)	3 945 (36)	<0.001
One or more limitations in IADL, *n* (%)	2 026 (7)	2 682 (24)	<0.001
One or more chronic diseases, *n* (%)	19 536 (71)	9 542 (86)	<0.001
One or more mobility limitations, *n* (%)	10 269 (37)	7 326 (66)	<0.001
Smoking, *n* (%)	18 997 (69)	7 762 (70)	0.02
Alcohol, *n* (%)	4 960 (18)	1 567 (14)	<0.001
Obesity^d^, *n* (%)	4 984 (18)	3 202 (29)	<0.001
Poor diet^e^, *n* (%)	6 032 (22)	2 950 (27)	<0.001

s.d., standard deviation; IADL, instrumental activities of daily living.

a International Standard Classification of Education level 5 or 6.

b Big city, its suburbs or outskirts.

c 4 or more points on EURO-D scale.

d Body mass index 30 and more.

e Fruits or vegetables less than every day.

Table 2 shows the results of the logistic regression models testing the association between PA trajectories and participants' sociodemographic and health-related characteristics. Compared with individuals with the constantly high PA, decreasing PA was associated with older age, lower education, lower household wealth, non-urban residence, not working, living without a partner, greater depressive symptoms, more limitations in IADL and mobility, more chronic diseases, smoking, obesity and poor diet. When compared to Western Europe, decreasing PA was more likely to occur in Southern Europe and Israel and less likely in Scandinavia and Central and Eastern Europe.

**Table 2.** Associations of participants’ characteristics with decreasing physical activity

Sociodemographic characteristics	OR (95% CI)
Age	1.08 (1.07–1.08)**
Women	1.01 (0.95–1.07)
High education^a^	0.60 (0.56–0.65)**
Highest decile of household net worth	0.61 (0.55–0.67)**
Urban residence^b^	0.94 (0.89–1.00)*
Employed or self-employed	0.70 (0.65–0.75)**
Two or more household members	1.05 (0.93–1.19)
Partner in household	0.82 (0.74–0.92)*
Two or more children	0.95 (0.89–1.01)
Two or more grandchildren	1.04 (0.98–1.11)
Region, *n* (%)	
Western Europe	Reference
Scandinavia	0.58 (0.53–0.65)**
Southern Europe	2.60 (2.41–2.79)**
Central and Eastern Europe	0.82 (0.76–0.88)**
Israel	1.84 (1.61–2.11)**
Health-related characteristics	
High depressive symptoms^c^	1.50 (1.42–1.60)**
One or more limitations in IADL	2.28 (2.12–2.46)**
One or more chronic diseases	1.28 (1.20–1.38)**
One or more mobility limitations	1.80 (1.70–1.91)**
Smoking	1.57 (1.48–1.66)**
Alcohol	0.95 (0.88–1.02)
Obesity^d^	1.63 (1.53–1.73)**
Poor diet^e^	1.44 (1.35–1.53)**

OR, odds ratio; CI, confidence interval.

All characteristics were entered into the model. In addition, the model included attrition and cognition (mean of z-scores of all three cognitive tests). Because of collinearity, birth cohort was not included into the model.

a International Standard Classification of Education level 5 or 6.

b Big city, its suburbs or outskirts.

c 4 or more points on EURO-D scale.

d Body mass index 30 and more.

e Fruits or vegetables less than every day.

*p < 0.05; **p < 0.001.

### Physical-activity trajectories and cognitive performance

Table 3 shows the results of the linear mixed-effects models. Compared with individuals from the constantly high PA group, individuals in the decreasing PA group had a significantly lower level of baseline CP, independently of all covariates (*p* < 0.001 for all three measures in Model 1, 2 and 3). Individuals in the decreasing PA group also had a steeper decline of CP in all CP measures, independently from covariates, as shown by the PA trajectory × time interaction (*p* < 0.001 for all three measures in Models 1, 2 and 3).

**Table 3.** Level of cognitive performance and rate of cognitive decline per year across trajectories of physical activity

	Model 1	Model 2	Model 3
	ß (95% CI)		
Association of decreasing physical activity with the level of cognitive performance			
Immediate recall	−0.47 (−0.50; −0.44)**	−0.18 (−0.21; −0.15)**	−0.10 (−0.13; −0.06)**
Verbal fluency	−2.58 (−2.73; −2.43)**	−1.03 (−1.17; −0.90)**	−0.71 (−0.85; −0.57)**
Delayed recall	−0.51 (−0.55; −0.47)**	−0.17 (−0.21; −0.14)**	−0.08 (−0.12; −0.04)**
Association of decreasing physical activity with the rate of cognitive decline (physical activity trajectory × time)			
Immediate recall	−0.03 (−0.04; −0.03)**	−0.03 (−0.04; −0.03)**	−0.03 (−0.04; −0.03)**
Verbal fluency	−0.17 (−0.19; −0.15)**	−0.15 (−0.17; −0.14)**	−0.16 (−0.17; −0.14)**
Delayed recall	−0.04 (−0.05; −0.04)**	−0.04 (−0.05; −0.04)**	−0.04 (−0.05; −0.04)**
Rate of cognitive decline stratified by the trajectory of physical activity			
Decreasing physical activity			
Immediate recall	−0.05 (−0.05; −0.04)**	−0.04 (−0.05; −0.04)**	−0.04 (−0.05; −0.04)**
Verbal fluency	−0.24 (−0.26; −0.23)**	−0.23 (−0.24; −0.21)**	−0.22 (−0.24; −0.21)**
Delayed recall	−0.05 (−0.05; −0.04)**	−0.04 (−0.05; −0.04)**	−0.04 (−0.05; −0.04)**
Constantly high physical activity			
Immediate recall	−0.01 (−0.01; −0.01)**	−0.01 (−0.01; −0.01)**	−0.01 (−0.01; −0.01)**
Verbal fluency	−0.07 (−0.08; −0.06)**	−0.06 (−0.06; −0.05) **	−0.05 (−0.06; −0.04)**
Delayed recall	−2.2 × 10^−3^ (−4.9 × 10^−3^; 5.4 × 10^−4^)	−2.2 × 10^−4^ (−2.5 × 10^−3^; 3.0 × 10^−3^)	6.0 × 10^−4^ (−2.2 × 10^−3^; 3.4 × 10^−3^)

CI, confidence interval.

Results are derived from linear mixed-effects models.

Model 1: adjusted for age and sex.

Model 2: adjusted for age, sex, birth cohort, region, education, residence, household size, a partner in household, household net worth, current job situation, number of children, number of grandchildren and attrition.

Model 3: adjusted for age, sex, birth cohort, region, education, residence, household size, a partner in household, household net worth, current job situation, number of children, number of grandchildren, attrition, limitations in IADL, depressive symptoms, number of chronic diseases, body mass index, mobility limitations index, smoking, alcohol use and eating behaviour.

*p < 0.05; **p < 0.001.

**Fig. 3.**
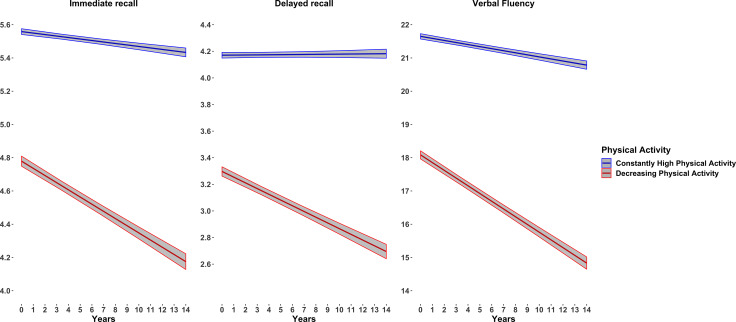
Crude yearly rates of cognitive decline across trajectories of physical activity.

When stratified by the PA trajectory, individuals from the constantly high PA group had negligible rates of decline in immediate recall, verbal fluency and none in delayed recall, while individuals from the decreasing PA group showed steeper rates of decline in all three cognitive measures (Fig. 3). Adjustment for covariates only slightly attenuated the rates of decline. In the fully adjusted models, individuals from the decreasing PA group had a steeper decline in all cognitive measures (immediate recall: ß = −0.04; 95% CI = −0.05 to −0.04; verbal fluency: ß = −0.22; 95% CI = −0.24 to −0.21; delayed recall: ß = −0.04; 95% CI = −0.05 to −0.04), in comparison to the other group. In contrast, individuals from the constantly high PA group only demonstrated a small decline in immediate recall (ß −0.01; 95% CI −0.01 to −0.01) and verbal fluency (ß = −0.05; 95% CI −0.06 to −0.04) and none in delayed recall (ß = 6.0 × 10^−4^; 95% CI −2.2 × 10^−3^ to 3.4 × 10^−3^).

### Sensitivity and robustness analyses

The SA and robustness analyses (Supplementary tables S4, S5 and S6) yielded similar results as the main analysis. Specifically, we replicated the 2-class solution (see supplementary methods). Furthermore, the linear mixed-effects models showed that, compared with individuals from the constantly high PA group, individuals from the decreasing PA group had a significantly lower baseline level of the three CP measures and exhibited a steeper decline of those measures. The only difference is that the associations between the PA groups and the decline of immediate and delayed recall over time did not remain significant after the adjustment for all the covariates.

## Discussion

In the present large-scale longitudinal multicentric study of adults aged 50 years and older, we identified two distinct PA trajectories: constantly high PA and decreasing PA. These profiles of PA trajectories were both associated with the level of CP and the rate of its decline over time. Compared with the constantly high PA profile, the decreasing PA profile was significantly associated with lower baseline levels of CP, and with a significantly steeper decline of CP over time. These associations were only slightly attenuated after adjustment for the sociodemographic and health-related characteristics. These findings suggest that the CP decline over time is mirrored in the longitudinal changes of PA.

Our results complement the literature on the association of PA and CP during ageing from a longitudinal perspective. Using growth mixture modelling, we identified two distinct PA trajectories over time. One PA trajectory showed PA levels that remained constant across time, a finding that is consistent with previous studies that observed stable PA trajectories in old age (Laddu *et al*., 2018; Aggio *et al*., 2020). The second PA trajectory that emerged showed a decreased PA across time. This result is in line with previous studies that have observed a reduction in PA at old age (Lounassalo *et al*., 2019; Sanchez-Sanchez *et al*., 2020). However, in addition to these two PA trajectories, other studies found increasing PA trajectories in middle-aged and older adults (Lounassalo *et al*., 2019; Saint-Maurice *et al*., 2019). Moreover, studies often reported three or four PA trajectory groups, which contrasts with the two PA trajectories that have emerged from the current data (Lounassalo *et al*., 2019). This discrepancy may be explained by the features of the scale that was used to measure PA (i.e., 2-items measuring the frequency of moderate and vigorous PA), which was associated with low variance. As such, it was difficult to identify clearly distinct PA trajectories as indicated by the low level of entropy observed between our two selected PA groups.

In the present study, 71% of our sample were classified in the more favourable profile (i.e., constantly high PA), while only 29% were classified in the unfavourable profile (i.e., decreasing PA). This result is rather consistent with previous studies observing that about 30% of older adults showed a decreasing PA trajectory across time (Saint-Maurice *et al*., 2019; Aggio *et al*., 2020; Sanchez-Sanchez *et al*., 2020). However, while this finding is very encouraging in terms of public health, it must be interpreted with caution given that, in our study, PA was assessed using a self-reported questionnaire, which may have biased the estimation of participants' PA behaviours. Furthermore, when age is used as the time scale instead of the wave of measurement (i.e., using an accelerated longitudinal design assessing PA evolution from 65 to 100 years), results showed a clear decline in PA across ageing in all the PA groups (Laddu *et al*., 2018, 2020).

We observed that a number of sociodemographic and health-related characteristics are associated with the PA trajectories. In particular, older age, worse socioeconomic status, poor lifestyle profile and worse health were associated with increased odds of belonging to the unfavourable PA group. Our findings are consistent with previous studies that have investigated the multiple correlates of higher engagement in PA (Trost *et al*., 2002; Bauman *et al*., 2012). For example, chronic conditions (Cheval *et al*., 2021), depressive symptoms (Choi *et al*., 2019), or lower-level education (Kirk and Rhodes, 2011), have been found to be associated with a lower level of engagement in PA. Our results confirm these associations but also reveal that these factors are not only associated with the level of PA engagement, but also with its evolution across time. Even though causality cannot be established due to the observational design of this study, these findings indicate that decline in PA and CP could be predicted. Focusing health care and preventive efforts on subgroups of individuals with low socioeconomic status and poor health profile could contribute to the prevention of decreasing PA and CP.

The strong relationship between higher levels of PA and better cognitive function are well-established (Cheval *et al*., 2018*b*, 2020*c*; Sebastiani *et al*., 2020), and our results confirm this association. To the best of our knowledge, our study was the first to investigate this association using a data-driven approach to identify PA trajectories. One study has estimated trajectories of PA over 28 years in people with or without dementia and found that PA started to decline 9 years before the diagnosis of dementia (Sabia *et al*., 2017). A result that rather suggests that changes in PA may simply result from the decline in CP, which is consistent with other studies that have observed an association from changes in CP to changes in PA (Cheval *et al*., 2020*c*). Moreover, intervention studies in older adults observed a protective effect of an increased PA on CP (Colcombe and Kramer, 2003; Angevaren *et al*., 2008), although other studies did not (Sink *et al*., 2015; Young *et al*., 2015). However, these studies focused on relatively short-term changes in PA (i.e., from one time of measurement to another one; or before and after an intervention), but disregarded the long-term trajectories of PA across time. Overall, our results are in line with previous studies showing that changes in PA are associated with changes in CP.

Several mechanisms have been suggested to explain the association between a higher level of PA and a maintained level of CP (Cotman and Berchtold, 2002; Colcombe and Kramer, 2003; Cotman *et al*., 2007; Hillman *et al*., 2008; Raichlen and Alexander, 2017). For example, PA has been associated with increased brain plasticity, angiogenesis, synaptogenesis and neurogenesis primarily through the release of growth factors such as brain-derived neurotrophic factor, insulin-like growth factor-1 and vascular endothelial growth factor (Cotman and Berchtold, 2002; Cotman *et al*., 2007; Hillman *et al*., 2008). Moreover, the cognitive demands inherent in many types of PA, which may include planning, reasoning, decision-making and multitasking, may also have positive effects on the brain (Raichlen and Alexander, 2017; Singh *et al*., 2019). Alternatively, additional mechanisms have been suggested to explain the association in the other direction – i.e., from cognitive functioning to higher engagement in physical activity (Cheval *et al*., 2018*a*, 2019, 2020*a*). In particular, anchored within the theory of effort minimisation in physical activity (TEMPA) (Cheval and Boisgontier, 2021), these studies showed that cognitive functions are critical to counteract the automatic tendency to effort minimisation, thereby favouring engagement in PA.

Among the strengths of the present study are the large sample size, the longitudinal design, the data-driven approach to identify PA trajectories across time and the reliance on three measures of CP based on established procedures. However, some limitations should be noted. First, PA was assessed using a self-reported questionnaire, which may have reduced measurement validity. Second, the two PA groups differed in both the initial PA levels (i.e., high *v.* moderate) and the evolution of PA across time (i.e., maintaining *v.* decreasing). As such, it was not possible to disentangle the influence of these two factors (i.e., level or slope) on CP. Future research should rely on a more detailed and reliable questionnaire or on device-based measures of PA to be able to better discriminate between PA trajectories, and their associations with CP. Likewise, as the PA groups differed regarding overall health-related condition, we cannot exclude that a steeper cognitive decline observed in the unfavourable PA profile can result from this poorer health condition rather than the change in PA. Furthermore, as it cannot be avoided in long-term longitudinal studies, we cannot exclude a selection bias due to attrition. Yet, to minimise this bias, our statistical analyses were adjusted for attrition and we conducted SA excluding participants who dropped out or died during the follow-up. Finally, the correlational nature of the SHARE design cannot guarantee causal links between PA trajectories and CP decline.

In conclusion, these findings confirm the hypothesis that an unfavourable PA profile may be associated with weaker CP in old age, thereby supporting the need to promote effective strategies to help individuals to maintain their PA levels over the life course.

## Data Availability

This SHARE dataset is available at http://www.share-project.org/data-access.html.

## Supplementary material

For supplementary material accompanying this paper visit http://doi.org/10.1017/S2045796021000688. click here to view supplementary material
