# Supplementary material for: Association between physical-activity trajectories and cognitive decline in adults 50 years of age or older
Source: Epidemiol Psychiatr Sci. 2021 Dec 27;30:e79. doi: 10.1017/S2045796021000688 (PMC8728586; doi:10.1017/S2045796021000688)
Supplement: Supplementary file 1 [file S2045796021000688sup001.docx]

**Supplemental material**

**Supplementary Methods.** Detailed information concerning the identification of the PA trajectory groups.

**Supplementary table S1.** Model parameters and results of the 2-, 3-, and 4-class latent basis growth mixture models.

**Supplementary table S2.** Class proportions and mean intercept and slope results in the 2 PA classes.

**Supplementary table S3.** Estimated slopes in the 2-class Growth Mixture Model with free time scores.

**Supplementary table S4.** Indicators of the models extracted for the robusticity and sensitivity analyses.

**Robustness analyses**

**Table S5.** Mixed-effects model results on the 2-class trajectories of moderate and vigorous physical activity measures

**Sensitivity analyses**

**Table S6.** Mixed-effects model results on the 2-class trajectories extracted on:

- Participants who had at least two measures of physical activity, two measures of cognitive performance, and not dementia diagnosis (N = 67 270)
- Participants who survived during the follow-up of the study (N=36 248)
- Participants who neither dropped out nor died during the follow-up of the study (N = 29 115)

**Supplementary figure S1a-c.** Mean physical activity over time across different numbers of classes extracted

**Supplementary figure S2a-e.** Results of the robusticity and sensitivity analyses

**Supplementary Methods.** Detailed information concerning the identification of the PA trajectory groups.

*Growth mixture modelling*

All the 2-, 3-, and 4-class models exhibited good fit, however, the 2-class model showed a relatively low entropy, indicating some overlap between the two classes (entropy = 0.603; see also Supplementary table S1, Supplementary figure S1a-c). The extracted two classes in the 2-class solution showed the following patterns: Class 1) Decreasing PA and Class 2) Constantly high physical activity (Supplementary tables S2-3). To test the robustness of the results we also replicated the analyses on the vigorous and moderate physical activity measures separately. Both measures showed a reliable 2-class model solution supported by all model indicators, but no further classes were possible to extract, due to the very low variance in the measures (Supplementary table S4 and Supplementary figure S2a-b). Thus, we chose the best model of PA-classes based on interpretability. We selected the 2-class model for further analyses, as the trajectories (i.e., constantly maintained activity vs decreasing activity) were essentially comparable across the vigorous activity, moderate activity, and combined PA measures, and extracting more classes was not supported neither by the separate vigorous and moderate activity measures nor by the interpretability of the 3-class PA solution (Supplementary figure S1b). The low entropy in the 2-class PA solution suggests, however, that the distinction for some of the class members is not clear.

Further, we performed sensitivity analyses on an extended sample involving participants having at least two measures of PA and cognition and not having dementia (N = 67 270). We also replicated the growth mixture modelling on a restricted subsample who did not die during the follow-up period of the study (N = 36 248); and on those who neither died nor dropped out from the study during the complete follow-up period (N = 29 115). The results of the robustness and sensitivity growth mixture modelling analyses can be found in Supplementary figure S2a‑e and Supplementary table S4. The results of the linear mixed-effects models in the robustness and sensitivity analyses can be found in Supplementary tables S5-6.

**Supplementary table S1.** Model parameters and results of the 2-, 3-, and 4-class latent basis growth mixture models.

|  | | | | | | | | |
| --- | --- | --- | --- | --- | --- | --- | --- | --- |
| **N of classes** | **Best Log-Likelihood** | **AIC** | **BIC** | **SSA BIC** | **Entropy** | **VLM RLR** | **LMR ALRT** | **BLRT** |
| 2 | -288450.981 | 576937.963 | 577092.121 | 577034.917 | 0.603 | p < 0.001 | p < 0.001 | p < 0.001 |
| 3 | -280226.384 | 560494.768 | 560674.619 | 560607.881 | 0.875 | p < 0.001 | p < 0.001 | p < 0.001 |
| 4 | -279067.952 | 558183.903 | 558389.448 | 558313.176 | 0.838 | p < 0.001 | p < 0.001 | p < 0.001 |
| *Note.* AIC=Akaike information criterion; BIC=Bayesian information criterion; SSA BIC=sample-size adjusted Bayesian information criterion; VLM RLT=Vuong-Lo-Mendell-Rubin likelihood ratio test; LMR ALRT=Luo-Mendell-Rubin adjusted likelihood ratio test; BLRT=bootstrap likelihood ratio test. | | | | | | | | |

**Supplementary table S2.** Class proportions and mean intercept and slope results in the 2 PA classes.

|  | | | | |
| --- | --- | --- | --- | --- |
|  | **N of class members** | **% of total N** | **Mean of the latent intercept factor (S.E.)** | **Mean of the latent slope factor (S.E.)** |
| **Class 1** | 11095 | 28.65% | 3.418 (0.056)*** | -1.863 (0.061)*** |
| **Class 2** | 27634 | 71.35% | 4.540 (0.027)*** | 0.000 (0.039) |
| S.E. = standard error of the mean. The slopes and the intercepts significantly differ between the two classes (p < 0.001).  *** p < 0.001. | | | | |

**Supplementary table S3.** Estimated slopes in the 2-class growth mixture model with free time scores.

|  | |
| --- | --- |
|  | **Estimates of free slopes (S.E.)** |
| **Physical Activity Wave 1** | 0.000 (0.000) |
| **Physical Activity Wave 2** | 0.186 (0.018)*** |
| **Physical Activity Wave 4** | 0.535 (0.018)*** |
| **Physical Activity Wave 5** | 0.755 (0.021)*** |
| **Physical Activity Wave 6** | 0.900 (0.021)*** |
| **Physical Activity Wave 7** | 1.000 (0.000) |
| S.E. = standard error of the mean.  *** p < 0.001. | |

| **Supplementary table S4.** Indicators of the models extracted for the robusticity and sensitivity analyses. | | | | | | | | |
| --- | --- | --- | --- | --- | --- | --- | --- | --- |
| **Model** | **Best Log-Likelihood** | **AIC** | **BIC** | **SSA BIC** | **Entropy** | **VLM RLR** | **LMR ALRT** | **BLRT** |
| 2-class model of vigorous activity^1^ | -222302.010 | 444640.021 | 444794.179 | 444736.975 | 0.891 | p < 0.001 | p < 0.001 | p < 0.001 |
| 2-class model of moderate activity^2^ | -181797.649 | 363631.299 | 363785.457 | 363728.253 | 0.933 | p < 0.001 | p < 0.001 | p < 0.001 |
| 2-class model on the extended sample^3^ | -403655.374 | 807346.749 | 807510.845 | 807453.641 | 0.599 | p < 0.001 | p < 0.001 | p < 0.001 |
| 2-class model on the sample restricted to surviving participants^4^ | -270045.459 | 540126.919 | 540279.885 | 540222.681 | 0.599 | p < 0.001 | p < 0.001 | p < 0.001 |
| 2-class model on the sample restricted to participants not dropping out^5^ | -223138.527 | 446313.055 | 446462.077 | 446404.873 | 0.610 | p < 0.001 | p < 0.001 | p < 0.001 |
| *Note.* AIC=Akaike information criterion; BIC=Bayesian information criterion; SSA BIC=sample-size adjusted Bayesian information criterion; VLM RLT=Vuong-Lo-Mendell-Rubin likelihood ratio test; LMR ALRT=Luo-Mendell-Rubin adjusted likelihood ratio test; BLRT=bootstrap likelihood ratio test.  ^1^ Extracting 2-class latent trajectories on the vigorous activity measures only (on the original sample, N = 38729).  ^2^ Extracting 2-class latent trajectories on the moderate activity measures only (on the original sample, N = 38729).  ^3^ Extended sample consisting of participants having at least 2 measures of PA, 2 measures of cognition, and no dementia, N = 67270. Extracting a third class could not yield a stable and replicable log-likelihood.  ^4^ Restricted sample to those who survived the whole follow-up period, N = 36248.  ^5^ Restricted sample to those who did not drop-out during the analysis (neither died nor opted out from the study), N = 29115. | | | | | | | | |

**Supplementary table S5.** Mixed-effects models of the robustness analyses

|  | **Model 1** | **Model 2** | **Model 3** |
| --- | --- | --- | --- |
|  | **ß (95% CI)** | | |
| **Moderate physical activity measure** | | | |
| Association of decreasing physical activity with the level of cognitive performance | | | |
| Immediate recall | -0.46 (-0.50; -0.42)** | -0.14 (-0.18; -0.11)** | -0.07 (-0.10; -0.03)** |
| Verbal fluency | -2.48 (-2.65; -2.30)** | -0.83 (-0.98; -0.68)** | -0.52 (-0.68; -0.37)** |
| Delayed recall | -0.46 (-0.51; -0.42)** | -0.09 (-0.13; -0.05)** | -0.01 (-0.05; 0.03)** |
| Association of decreasing physical activity with the rate of cognitive decline (physical activity  trajectory × time) | | | |
| Immediate recall | -0.04 (-0.04; -0.03)** | -0.04 (-0.04; -0.03)** | -0.04 (-0.04; -0.03)** |
| Verbal fluency | -0.17 (-0.19; -0.15)** | -0.16 (-0.17; -0.14)** | -0.16 (-0.18; -0.14)** |
| Delayed recall | -0.05 (-0.05; -0.04)** | -0.04 (-0.05; -0.04)** | -0.04 (-0.05; -0.04)** |
| **Vigorous physical activity measure** | | | |
| Association of decreasing physical activity with the level of cognitive performance | | | |
| Immediate recall | -0.30 (-0.33; -0.27)** | -0.05 (-0.08; -0.03)** | -0.01 (-0.04; 0.01) |
| Verbal fluency | -1.79 (-1.92; -1.65)** | -0.53 (-0.65; -0.41)** | -0.37 (-0.49; -0.25)** |
| Delayed recall | -0.34 (-0.38; -0.31)** | -0.05 (-0.08; -0.02)* | -0.01 (-0.04; 0.03) |
| Association of decreasing physical activity with the rate of cognitive decline (physical activity  trajectory × time) | | | |
| Immediate recall | -0.03 (-0.03; -0.02)** | -0.02 (-0.03; -0.02)** | -0.03 (-0.03; -0.02)** |
| Verbal fluency | -0.11 (-0.13; -0.09)** | -0.10 (-0.12; -0.09)** | -0.10 (-0.12; -0.09)** |
| Delayed recall | -0.04 (-0.04; -0.03)** | -0.04 (-0.04; -0.03)** | -0.04 (-0.04; -0.03)** |
| **p<0.001  CI, confidence interval  Results are derived from linear mixed effects models.  Model 1: adjusted for age and sex  Model 2: adjusted for age, sex, birth cohort, region, education, residence, household size, partner in household, household net worth, current job situation, number of children, number of grandchildren and attrition  Model 3: adjusted for age, sex, birth cohort, region, education, residence, household size, partner in household, household net worth, current job situation, number of children, number of grandchildren, attrition, limitations in instrumental activities of daily living, depressive symptoms, number of chronic diseases, body mass index, mobility limitations index, smoking, alcohol use and eating behavior | | | |

**Supplementary table S6.** Mixed-effects models of the sensitivity analyses

|  | **Model 1** | **Model 2** | **Model 3** |
| --- | --- | --- | --- |
|  | **ß (95% CI)** | | |
| **Participants who had at least two measures of physical activity, two measures of cognitive performance, and no dementia diagnosis (N = 67 270)** | | | |
| Association of decreasing physical activity with the level of cognitive performance | | | |
| Immediate recall | -0.54 (-0.56; -0.51) | -0.22 (-0.25; -0.20)** | -0.14 (-0.16; -0.11)** |
| Verbal fluency | -2.91 (-3.02; -2.79)** | -1.27 (-1.37; -1.17)** | -0.93 (-1.04; -0.82)** |
| Delayed recall | -0.57 (-0.60; -0.54)** | -0.21 (-0.23; -0.18)** | -0.11 (-0.14; -0.08)** |
| Association of decreasing physical activity with the rate of cognitive decline (physical activity  trajectory × time) | | | |
| Immediate recall | -0.03 (-0.03; -0.03)** | -0.03 (-0.03; -0.02)** | -0.03 (-0.03; -0.03)** |
| Verbal fluency | -0.15 (-0.16; -0.13)** | -0.13 (-0.15; -0.12)** | -0.14 (-0.16; -0.12)** |
| Delayed recall | -0.04 (-0.04; -0.04)** | -0.04 (-0.04; -0.03)** | -0.04 (-0.04; -0.03)** |
| **Participants who survived during the follow-up of the study (N = 36 248)** | | | |
| Association of decreasing physical activity with the level of cognitive performance | | | |
| Immediate recall | -0.45 (-0.48; -0.42)** | -0.15 (-0.18; -0.12)** | -0.09 (-0.12; -0.05)** |
| Verbal fluency | -2.58 (-2.73; -2.42)** | -0.95 (-1.08; -0.81)** | -0.68 (-0.82; -0.54)** |
| Delayed recall | -0.50 (-0.54; -0.46)** | -0.14 (-0.18; -0.10)** | -0.07 (-0.11; -0.03)** |
| Association of decreasing physical activity with the rate of cognitive decline (physical activity  trajectory × time) | | | |
| Immediate recall | -0.03 (-0.04; -0.03)** | -0.03 (-0.03; -0.03)** | -0.03 (-0.03; -0.03)** |
| Verbal fluency | -0.15 (-0.17; -0.13)** | -0.14 (-0.16; -0.12)** | -0.14 (-0.16; -0.13)** |
| Delayed recall | -0.04 (-0.05; -0.04)** | -0.04 (-0.04; -0.03)** | -0.04 (-0.04; -0.03)** |
| **Participants who neither dropped out nor died during the follow-up of the study (N = 29 115)** | | | |
| Association of decreasing physical activity with the level of cognitive performance | | | |
| Immediate recall | -0.45 (-0.49; -0.42)** | -0.14 (-0.18; -0.11)** | -0.08 (-0.11; -0.05)** |
| Verbal fluency | -2.61 (-2.78; -2.43)** | -0.95 (-1.10; -0.80)** | -0.69 (-0.84; -0.53)** |
| Delayed recall | -0.52 (-0.56; -0.47)** | -0.15 (-0.19; -0.11)** | -0.08 (-0.12; -0.04)** |
| Association of decreasing physical activity with the rate of cognitive decline (physical activity  trajectory × time) | | | |
| Immediate recall | -0.03 (-0.04; -0.03)** | -0.03 (-0.03; -0.02)** | -0.03 (-0.03; -0.02)** |
| Verbal fluency | -0.17 (-0.18; -0.15)** | -0.15 (-0.17; -0.13)** | -0.15 (-0.17; -0.13)** |
| Delayed recall | -0.04 (-0.05; -0.03)** | -0.04 (-0.04; -0.03)** | -0.04 (-0.04; -0.03)** |
| **p<0.001  CI, confidence interval  Results are derived from linear mixed effects models.  Model 1: adjusted for age and sex  Model 2: adjusted for age, sex, birth cohort, region, education, residence, household size, partner in household, household net worth, current job situation, number of children, number of grandchildren and attrition  Model 3: adjusted for age, sex, birth cohort, region, education, residence, household size, partner in household, household net worth, current job situation, number of children, number of grandchildren, attrition, limitations in instrumental activities of daily living, depressive symptoms, number of chronic diseases, body mass index, mobility limitations index, smoking, alcohol use and eating behavior | | | |

| **a**  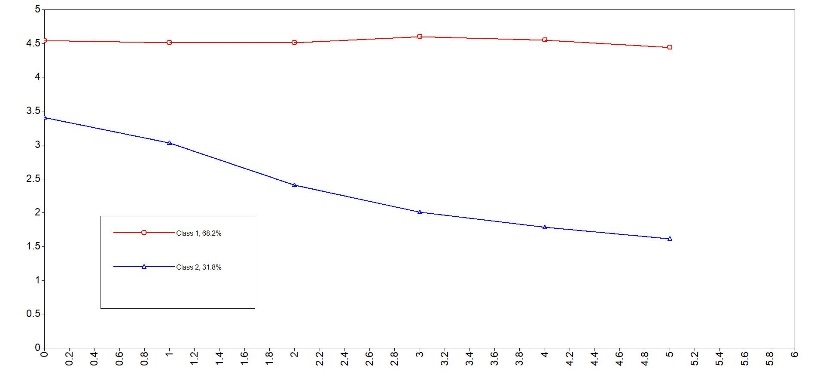 | **b**  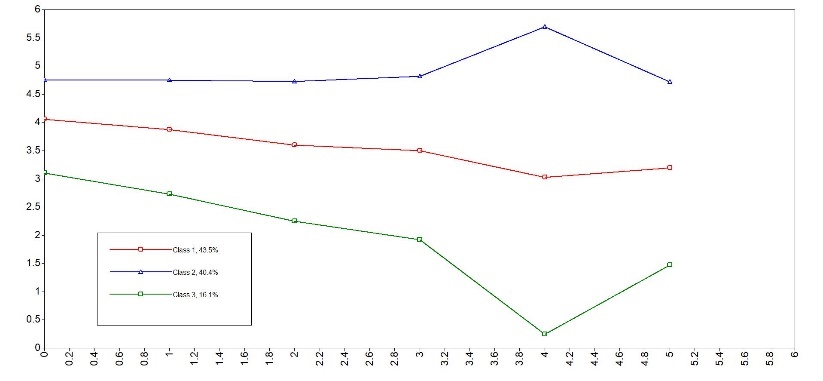 |
| --- | --- |
| **c**  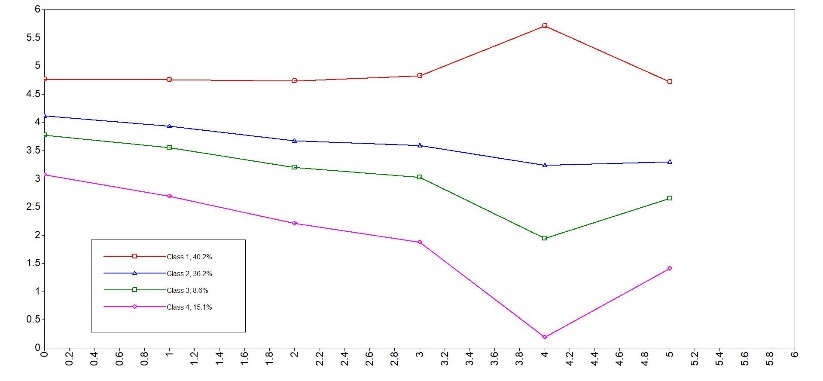 |  |

**Supplementary figure S1a-c** Mean physical activity (PA) over time across different numbers of classes extracted

**a** 2‑class physical activity model; **b** 3‑class physical activity model; **c** 4‑class physical activity model

| **a**  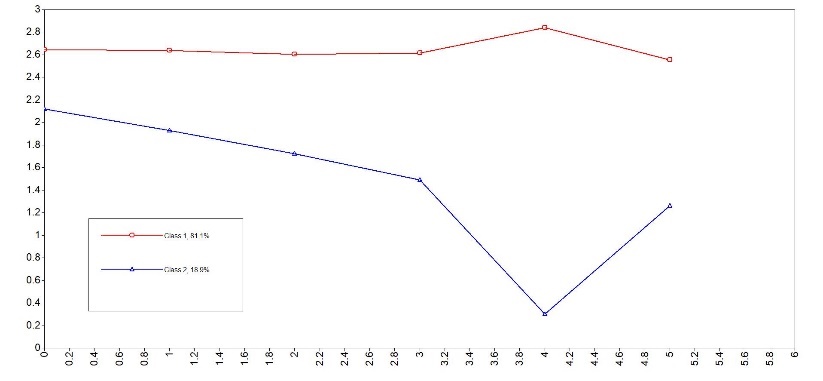 | **b**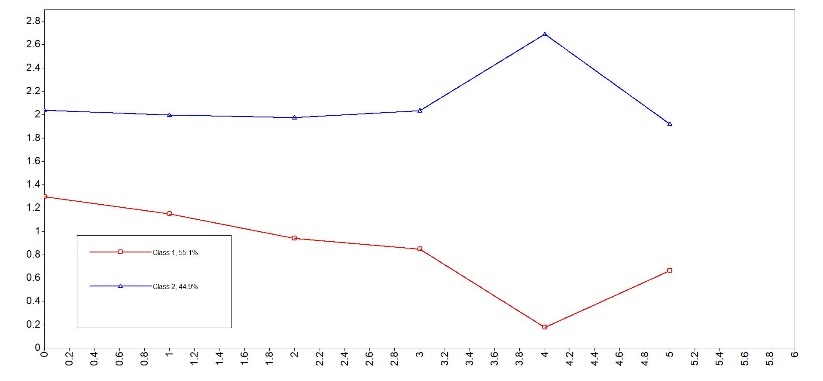 |
| --- | --- |
| **c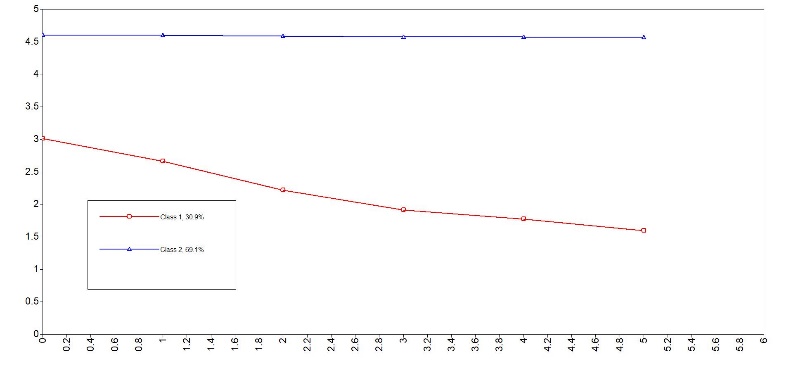** | **d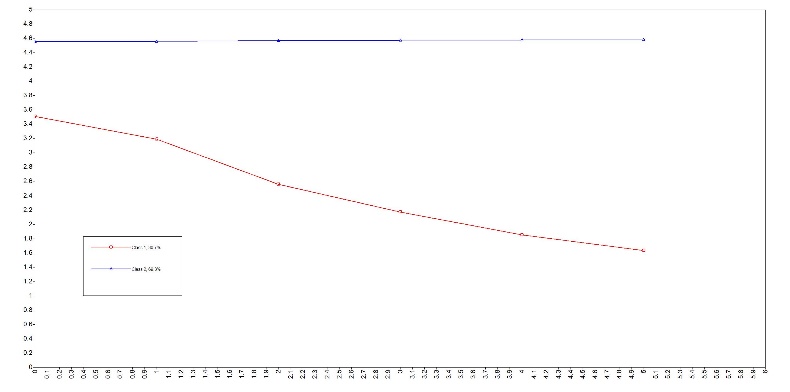** |
| **e**  **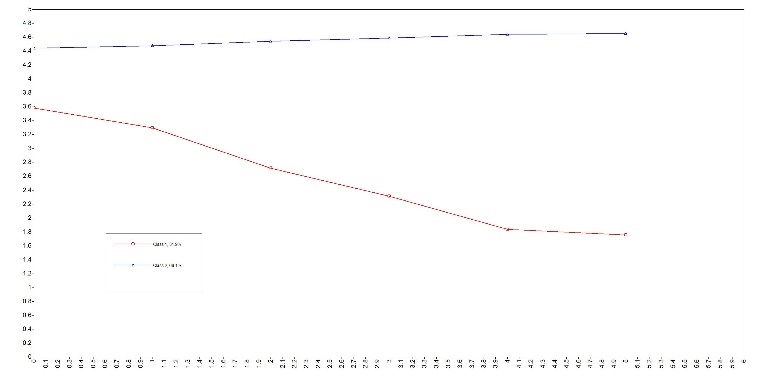** |  |

**Supplementary figure S2a-e.** Results of the robusticity and sensitivity analyses

**a** 2-class results extracting trajectories of moderate; **b** and vigorous activity separately instead of the pooled PA measure; **c** 2-class model on an extended sample (N = 67270); **d** 2-class model restricted to surviving participants (N = 36248); **e** 2-class model restricted to participants who did not drop out neither died (N = 29115). See the details of the models in the Supplementary table S4.
